# Improving Image Quality of On-Board Cone-Beam CT in Radiation Therapy Using Image Information Provided by Planning Multi-Detector CT: A Phantom Study

**DOI:** 10.1371/journal.pone.0157072

**Published:** 2016-06-09

**Authors:** Ching-Ching Yang, Fong-Lin Chen, Yeh-Chi Lo

**Affiliations:** 1 Department of Medical Imaging and Radiological Sciences, Tzu-Chi University of Science and Technology, Hualien, Taiwan; 2 Department of Medical Physics, Koo Foundation Sun Yat-Sen Cancer Center, Taipei City, Taiwan; 3 Department of Radiation Oncology, Mount Sinai Medical School, New York, New York, United States of America; North Shore Long Island Jewish Health System, UNITED STATES

## Abstract

**Purpose:**

The aim of this study was to improve the image quality of cone-beam computed tomography (CBCT) mounted on the gantry of a linear accelerator used in radiation therapy based on the image information provided by planning multi-detector CT (MDCT).

**Methods:**

MDCT-based shading correction for CBCT and virtual monochromatic CT (VMCT) synthesized using the dual-energy method were performed. In VMCT, the high-energy data were obtained from CBCT, while the low-energy data were obtained from MDCT. An electron density phantom was used to investigate the efficacy of shading correction and VMCT on improving the target detectability, Hounsfield unit (HU) accuracy and variation, which were quantified by calculating the contrast-to-noise ratio (CNR), the percent difference (%Diff) and the standard deviation of the CT numbers for tissue equivalent background material, respectively. Treatment plan studies for a chest phantom were conducted to investigate the effects of image quality improvement on dose planning.

**Results:**

For the electron density phantom, the mean value of CNR was 17.84, 26.78 and 34.31 in CBCT, shading-corrected CBCT and VMCT, respectively. The mean value of %Diff was 152.67%, 11.93% and 7.66% in CBCT, shading-corrected CBCT and VMCT, respectively. The standard deviation within a uniform background of CBCT, shading-corrected CBCT and VMCT was 85, 23 and 15 HU, respectively. With regards to the chest phantom, the monitor unit (MU) difference between the treatment plan calculated using MDCT and those based on CBCT, shading corrected CBCT and VMCT was 6.32%, 1.05% and 0.94%, respectively.

**Conclusions:**

Enhancement of image quality in on-board CBCT can contribute to daily patient setup and adaptive dose delivery, thus enabling higher confidence in patient treatment accuracy in radiation therapy. Based on our results, VMCT has the highest image quality, followed by the shading corrected CBCT and the original CBCT. The research results presented in this study should be able to provide a route to reach a high level of image quality for CBCT imaging in radiation oncology.

## Introduction

During a course of radiotherapy treatment, any displacement of the target region leads to a lowered dose being delivered to the target. Only once the accuracy of dose delivered to a target volume is established can radiotherapy further improve treatment outcomes through target dose escalation or normal tissue sparing [1, 2]. In modern radiation therapy, patients are scanned by multi-detector CT (MDCT) for treatment planning purposes, which can be limited due to changes in the patient’s anatomy and the extent of the tumor during daily treatment. Cone-beam CT (CBCT) mounted to the linear accelerator allows the acquisition of a CT scan of the patient in the treatment position, so it could reduce patient set-up errors and organ displacement during the course of treatment [1, 3, 4]. In CBCT-based image guided radiotherapy (IGRT), image contrast must be adequate to resolve target structures of both high- and low-contrast objects as CBCT images acquired after treatment setup are used to ensure accurate localization of the target or to ensure that the target is in the planned position relative to the treatment image. Hounsfield unit (HU) fidelity is also important if the CBCT images will be used for dose calculation. In spite of the increasing use of CBCT for patient set-up verification, the shading artifacts in CBCT which occur due to increased photon scattering, beam hardening effects and background variations cause significant variability in HU values [5–8]. Hence, the CT numbers of CBCT images do not have a unique, one-to-one relationship with tissue densities and pose challenges to its use in adaptive radiotherapy (ART) [9, 10]. It has been reported that using a fixed CT-to-density conversion table can lead to errors in the heterogeneous dose calculations on CBCT scans ranging from 1% to 5% [11–13]. On the other hand, MDCT scanners provide accurate image information because of small inherent scanner signals, as well as more linear detectors and sophisticated correction algorithms that have been developed over the past several decades [9, 10, 14]. Several studies have investigated the use of MDCT images as prior information to improve the image quality of CBCT [11, 12]. Marchant et al. have proposed a shading correction method which enhances CBCT scans by a low spatial frequency grey scale shading function generated with the aid of MDCT scan [14]. Their method can effectively increase the accuracy of CBCT density values, but it cannot estimate high-frequency statistical error and scatter noise. The purpose of this study was to improve the image quality of on-board CBCT in terms of target detectability and HU fidelity based on the image information provided by planning MDCT. To reach this goal, a virtual monochromatic CT (VMCT) synthesized using CBCT + MDCT was performed and evaluated using two phantom studies.

## Materials and Methods

### CBCT and MDCT scans

The CBCT scans were performed using the on-board imager system installed on the Varian TrueBeam radiation therapy machine (Varian Medical System, Palo Alto, CA, USA). In the full-fan scan mode (field of view = 26.17 cm), the detector is centered laterally and longitudinally with respect to the source. In the half-fan scan mode (field of view = 46.5 cm), the detector is shifted laterally 16 cm. The torso CBCT protocols used 1070 mAs at 125 kVp with weighted CT dose index (CTDI_w_) of 14.3 mGy. The CBCT image acquired in half-fan mode and reconstructed using manufacturer’s software has a size of 512 × 512 with voxel size of 0.9085 mm in the axial plane and slice thickness of 1.987 mm. The MDCT scans were taken on a 16-slice Philips Brilliance Big Bore CT simulator (Philips Healthcare Systems, Andover, MA, USA) in axial scan mode with 16 × 1.5 mm collimation. The torso MDCT protocols used 500 mAs at 120 kVp with volume CT dose index (CTDI_vol_) of 55.9 mGy, defined as the standard protocol in this study. A low-energy MDCT protocol with 90 kVp and 500 mAs was also performed (Table 1). The MDCT image reconstructed using manufacturer’s software has a size of 512 × 512, with voxel size of 1.17 mm in the axial plane and 3 mm in the longitudinal direction.

**Table 1:** Imaging parameters of the CBCT and MDCT systems.

**CBCT**	**Tube voltage**	**Tube current-time product**
Standard (CTDI_w_ = 14.3 mGy)	125 kVp	1070 mAs (80 mA, 13.38 sec)
**MDCT**	**Tube voltage**	**Tube current-time product**
Low energy (CTDI_vol_ = 25.4 mGy)	90 kVp	500 mAs (286 mA, 1.75 sec)
Standard (CTDI_vol_ = 55.9 mGy)	120 kVp	500 mAs (286 mA, 1.75 sec)

### MDCT-based shading correction for CBCT

The MDCT-based shading correction method proposed by Marchant et al. was used in this study [14]. The method hypothesizes that scatter signals as well as many beam hardening and lag-related artifacts have dominant low-frequency components, so only slowly varying differences between MDCT and CBCT are to be corrected. The shading correction begins with an affine registration between MDCT and CBCT to calculate a ratio image that indicates the difference between the two images. Next, the ratio image was smoothed using a low-pass 2D Gaussian filter with a window size of 21-by-21 pixels and a standard deviation of 11 pixels. The smoothed ratio image was subsequently used to correct the CBCT by division. On a 3.4 GHz PC, the affine registration and the shading correction take about 10 minutes and 1 minute in MATLAB 7.1 (The Mathworks, Natick, MA, USA), respectively.

### Virtual monochromatic CT image

**Fig 1:**
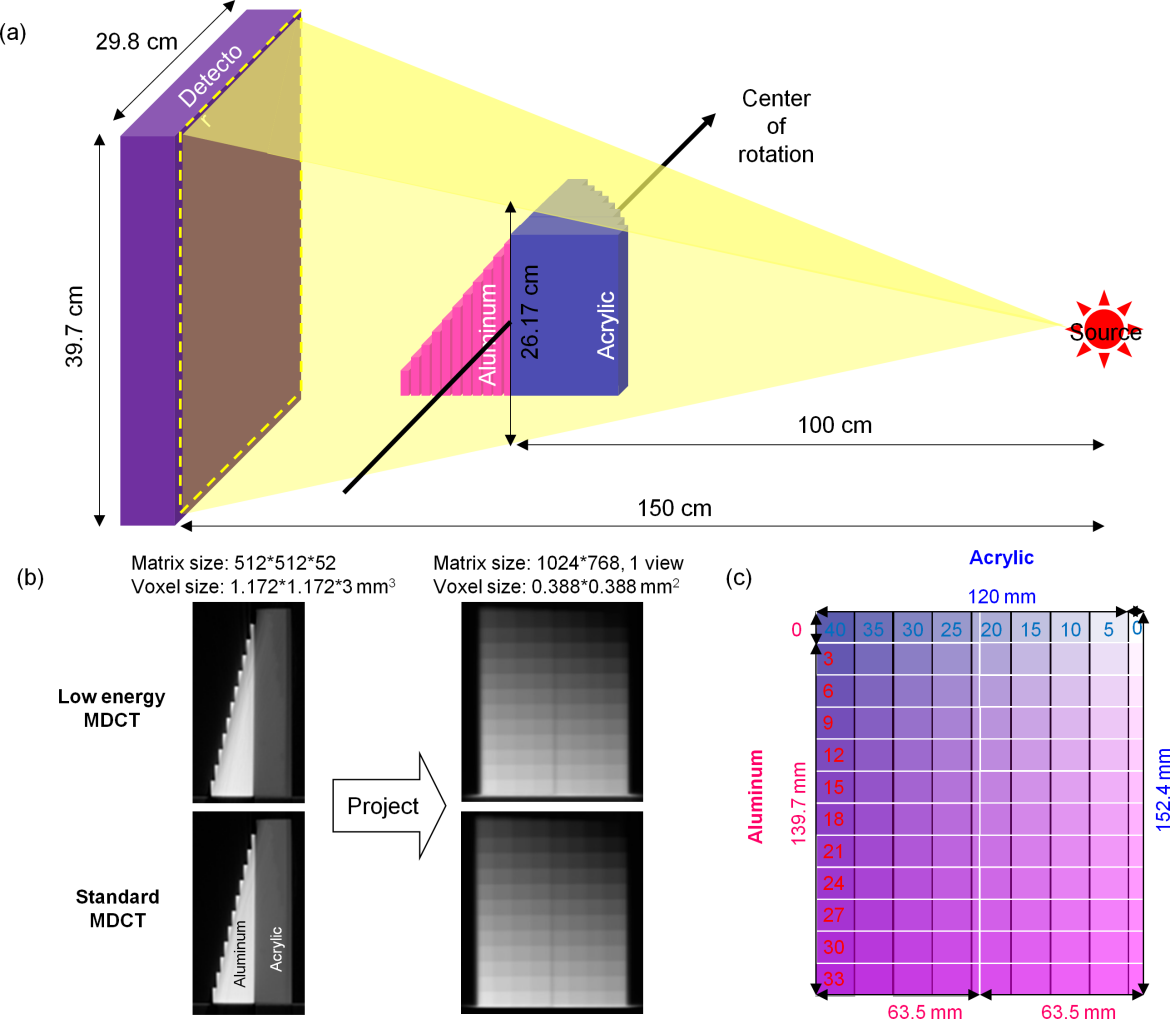
(a) Illustration of the calibration step wedge setup in relation to the geometry of CBCT to forward project axial images into projections. (b) Axial images of the calibration step wedge acquired using MDCT (left) and the corresponding projections obtained via forward projection (right). (c) Illustration of the calibration step wedge setup relative to the projection.

In this study, the VMCT technique proposed by Li et al [15] was used. Basically, the workflow includes: (1) the decomposition of CT projection data into the equivalent thickness of two basis materials and (2) the linear combination of density maps to synthesize virtual monochromatic images. Aluminum and acrylic were used as the basis materials to simulate bone and soft tissue, respectively. In the decomposition step, low-energy projections (L) and high-energy projections (H) are used to estimate the equivalent thicknesses of aluminum (x_A_) and acrylic (x_B_):

$$x_{A}=\frac{a_{0}+a_{1}L+a_{2}H+a_{3}L^{2}+a_{4}LH+a_{5}H^{2}}{1+b_{0}L+b_{1}H}$$ (1)

$$x_{B}=\frac{c_{0}+c_{1}L+c_{2}H+c_{3}L^{2}+c_{4}LH+c_{5}H^{2}}{1+d_{0}L+d_{1}H},$$ (2)

where the parameters a_i_, b_j_ c_i_, d_j_ (i = 0–5; j = 0, 1) represent characteristics of the x-ray beam energy spectrum. In the combination step, virtual monochromatic projections were synthesized using the following equation:

$$\int \mu (E)ds=\mu_{A}(E)x_{A}+\mu_{B}(E)x_{B},$$ (3)

where μ_A_(E) and μ_B_(E) are the linear attenuation coefficients of basis materials at energy E. Before performing VMCT, the parameters a_i_, b_j_ c_i_, d_j_ in Eqs 1 and 2 need to be determined through a calibration experiment using aluminum and acrylic step wedges stacked in an orthogonal pattern. This step is called parameterization, and is the only step that needs to know the material composition of the object being scanned. The aluminum step wedge with 11 steps (Fluke Biomedical, Everett, WA, USA) is 139.7 mm in length, 63.5 mm in width and 33 mm in heighy. The home-made acrylic step wedge with 8 steps is 120 mm in length, 152.4 mm in width and 40 mm in height. Images reconstructed in mm^-1^ of the calibration step wedge were acquired with low-energy and standard MDCT protocols and were then forward projected to obtain projections at two different energies based on Siddon’s ray tracing algorithm (Fig 1) [16]. Forty eight ROIs were placed on the projections to determine L and H used in Eqs 1 and 2 (x_A_ = 0, 6, 12, 18, 24, 30 mm; x_B_ = 5, 10, 15, 20, 25, 30, 35, 40 mm). Given L, H, x_A_ and x_B_ of the calibration step wedge, the parameters a_i_, b_j_ c_i_, d_j_ can be determined by minimizing absolute error fitting. To validate the results of parameterization, the thicknesses of aluminum and acrylic step wedges estimated based on Eqs 1 and 2 were compared with those measured using a caliber.

**Fig 2:**
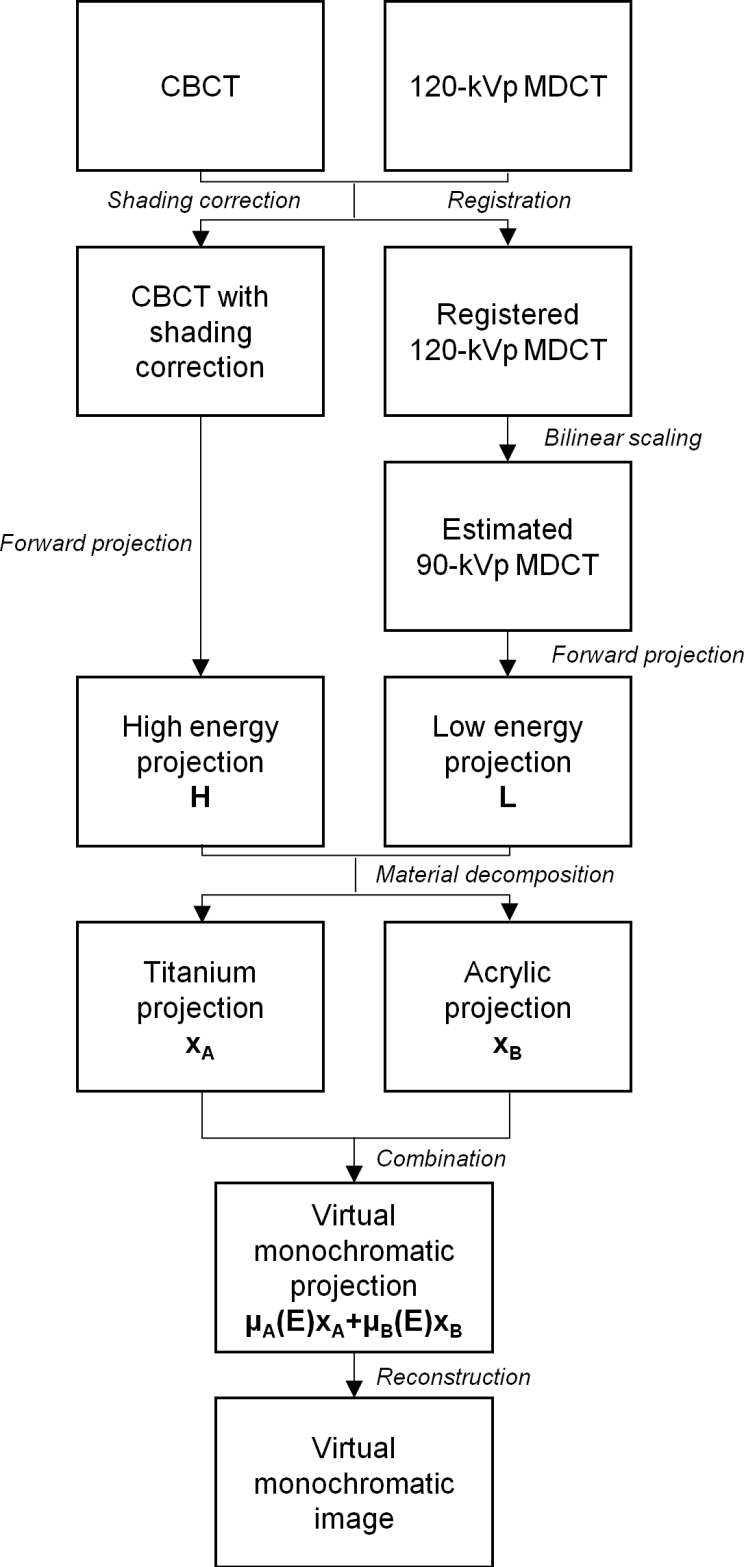
Workflow of VMCT images synthesized using CBCT + MDCT.

**Fig 3:**
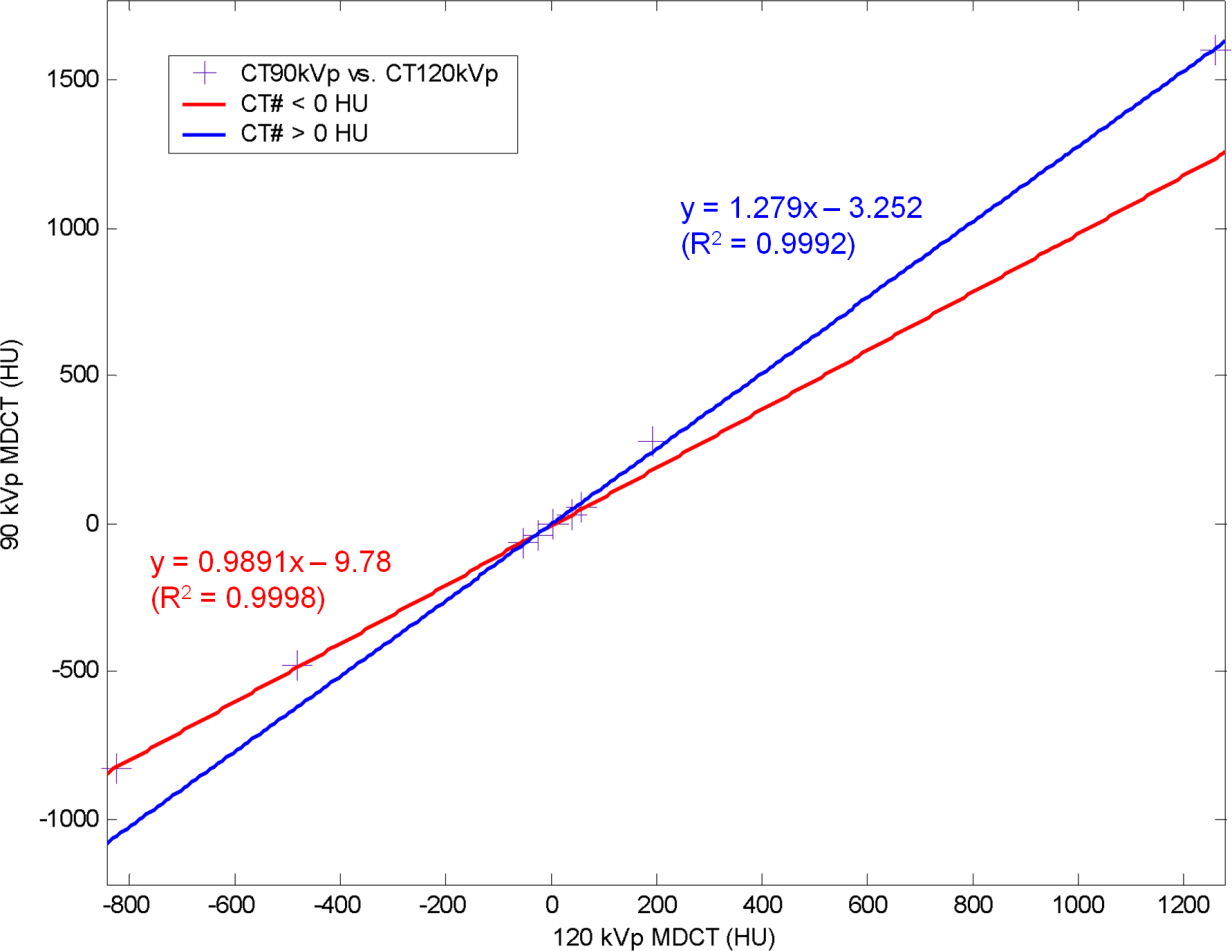
The bilinear scaling functions used to convert 120-kVp MDCT to 90-kVp MDCT. The R^2^ is the coefficient of determination.

After parameterization, VMCT can be synthesized by conducting the decomposition and the combination steps for any object scanned with the same imaging protocols as those used in the calibration step wedge experiment. In the results reported by Marchant et al., the shading corrected CBCT agreed with the HU values in MDCT within 1% for soft tissue regions (fat and muscle) and showed improved agreement for the bone region, which inspired us to evaluate the feasibility of synthesizing VMCT using CBCT + MDCT instead of using low-energy MDCT + standard MDCT. Fig 2 shows the proposed workflow to synthesize VMCT based on CBCT + MDCT. First, reconstructed images were acquired using CBCT and standard MDCT protocols. Next, the MDCT-based shading correction proposed by Marchant et al. was performed to improve the accuracy of CBCT density value. The corrected CBCT images were forward projected to obtain the high-energy projections H. On the other hand, the MDCT images were registered with CBCT images by using an affine transformation and then converted to 90-kVp MDCT based on the bilinear scaling method [17]. The bilinear calibration curves were obtained by relating HUs in 120-kVp MDCT of the electron density phantom to the corresponding HUs in 90-kVp MDCT (Fig 3), where the HUs in 120-kVp MDCT images were divided into two regions (< 0 HU and ≥ 0 HU). The experimentally derived conversion equations were used to convert MDCT energy from 120 kVp to 90 kVp. The estimated 90-kVp MDCT images were then forward projected to obtain the low-energy projections L. Next, aluminum (x_A)_ and acrylic (x_B_) projections in mm were estimated using Eqs 1 and 2 on a pixel by pixel basis. The decomposed projections were then used to synthesize monochromatic projections based on Eq 3. The mass attenuation coefficients of aluminum and acrylic at different energies were obtained from XCOM: Photon Cross Sections Database by National Institute of Standards and Technology (NIST), available at http://physics.nist.gov/xcom. A standard FDK algorithm was used for the VMCT reconstruction. The CBCT forward projector, FDK reconstruction and other image processing tools were implemented in MATLAB 7.1. On a 3.4 GHz PC, the virtual monochromatic projections take about 25 seconds per view, and the image reconstruction takes about 2 minutes. To verify the proposed workflow, the absolute difference between VMCT images synthesized using low-energy MDCT + standard MDCT and those using CBCT + MDCT was calculated for comparison purpose.

### Phantom study I: Electron density phantom

**Fig 4:**
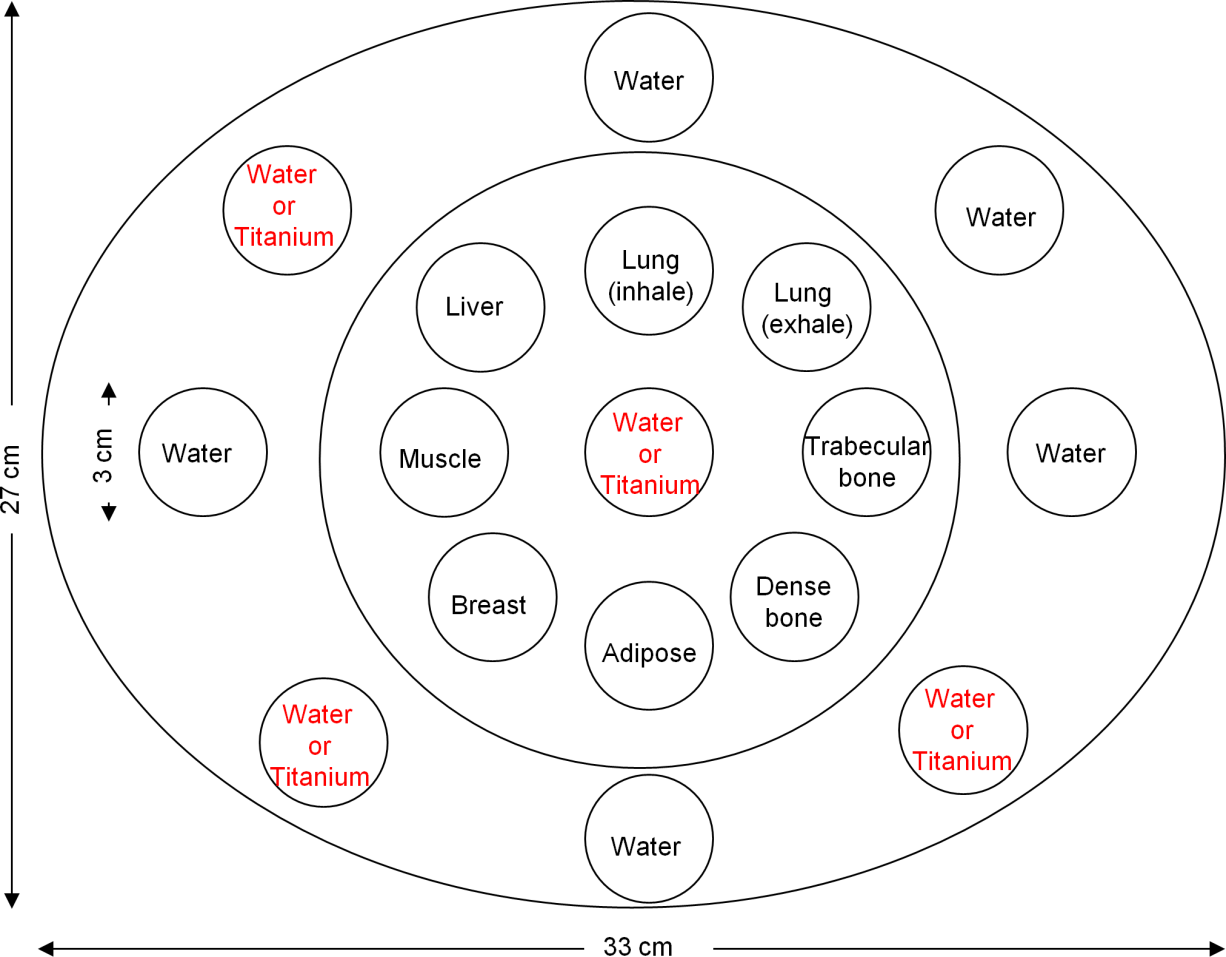
Illustration of the electron density phantom.

**Fig 5:**
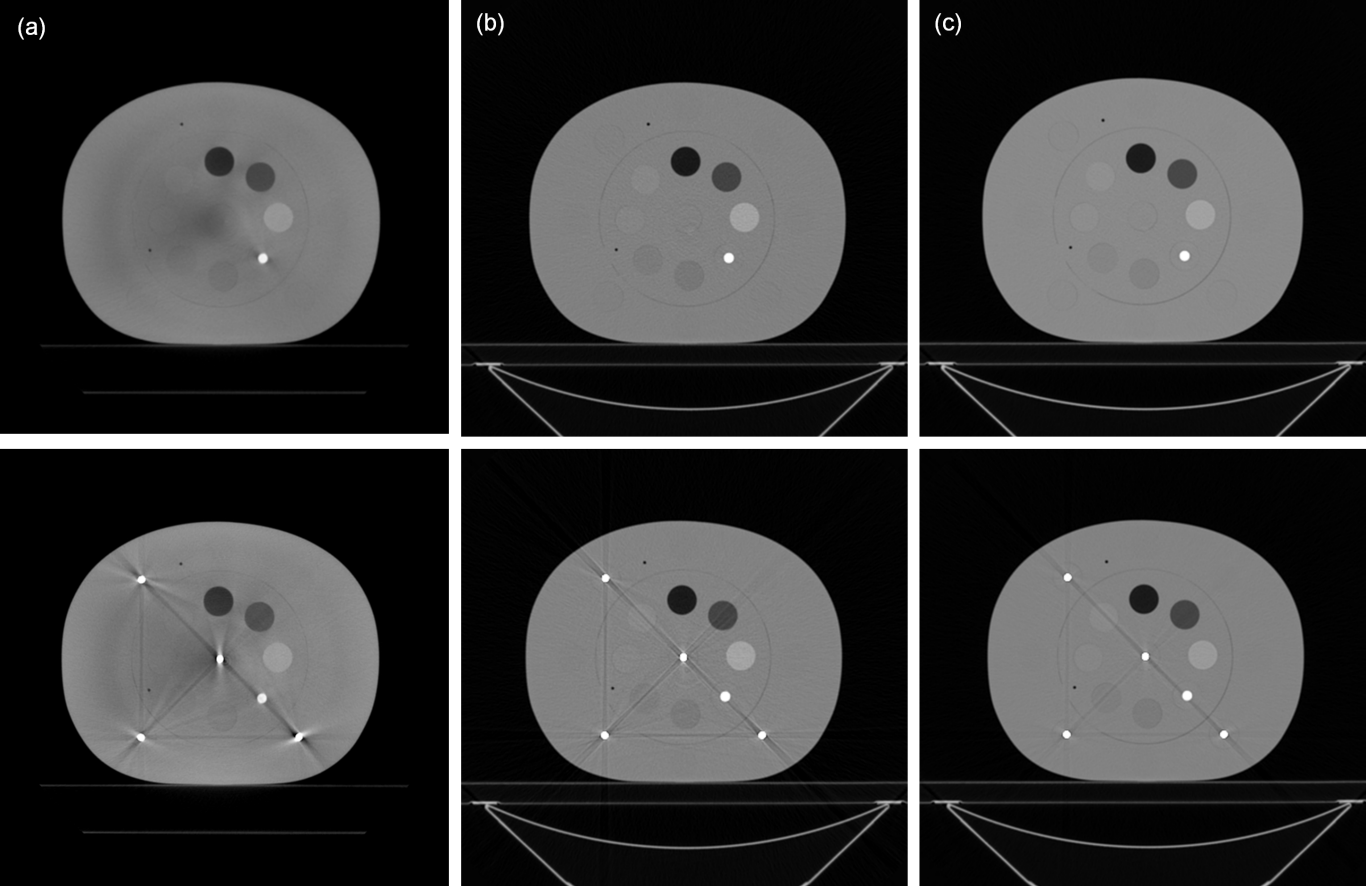
Axial image of the electron density phantom without (top row) and with (bottom row) titanium inserts acquired using (a) CBCT, (b) low-energy MDCT and (c) standard MDCT.

The electron density phantom (Model 062; CIRS, Norfolk, VA, USA) drilled with 17 holes was made of soft tissue equivalent epoxy resin (Fig 4). The overall dimensions of the phantom are 33 cm × 22 cm × 5 cm, which can be configured to simulate torso setup. Rod inserts simulating lung (inhale: 0.20 g/cc; exhale: 0.50 g/cc), trabecular bone (1.16 g/cc), dense bone (1.53 g/cc), adipose (0.96 g/cc), breast (0.99 g/cc), muscle (1.06 g/cc), liver (1.07 g/cc) and plastic water (1.01 g/cc) were inserted into the holes. Scanning was performed using the electron density phantom without and with titanium inserts in 4 different holes (Fig 5). Rod inserts are 3 cm in diameter and 5 cm in height, whereas the dense bone and titanium inserts have a core (dense bone: 1 cm in diameter; titanium insert: 0.6 cm in diameter) surrounded by soft tissue equivalent epoxy resin. A circular region-of-interest (ROI) of 40 pixels was placed on the soft tissue equivalent epoxy resin (background) and the rod inserts simulating different tissue materials (target region) to calculate the mean and standard deviation of HU within ROI. Because target detection is dependent on both image contrast and image noise, it was quantified by calculating the contrast-to-noise ratio (CNR):

$$CNR=\left|\frac{CT\#-{CT\#}_{BG}}{{SD}_{BG}}\right|$$ (4)

where CT# is the mean CT number of a specified material, CT#_BG_ and SD_BG_ are the average and standard deviation of CT numbers of tissue equivalent background material, respectively. The HU values in MDCT have one-to-one relationship with tissue densities, so MDCT allows accurate dose calculation through the transfer of HU values to electron densities. For CBCT, shading corrected CBCT and VMCT, the HU accuracy and variation were evaluated since they determine the HU-to-density conversion relationship, and may thus affect the dose calculation accuracy in treatment planning. The HU accuracy was monitored for rod inserts simulating different tissue materials by calculating the percent difference (%Diff):

$$\%Diff\;(\%)=\left|\frac{CT\#-{CT\#}_{MDCT}}{{CT\#}_{MDCT}}\right|∙100$$ (5)

where CT# is the mean CT number of a specified material in CBCT, shading corrected CBCT or VMCT, CT#_MDCT_ is the mean CT number in MDCT for the same tissue material. With regards to the variability in HU values, the standard deviation of the CT numbers for tissue equivalent background material within a ROI of 10720 pixels was calculated.

### Phantom study II: Anthropomorphic chest phantom

**Fig 6:**
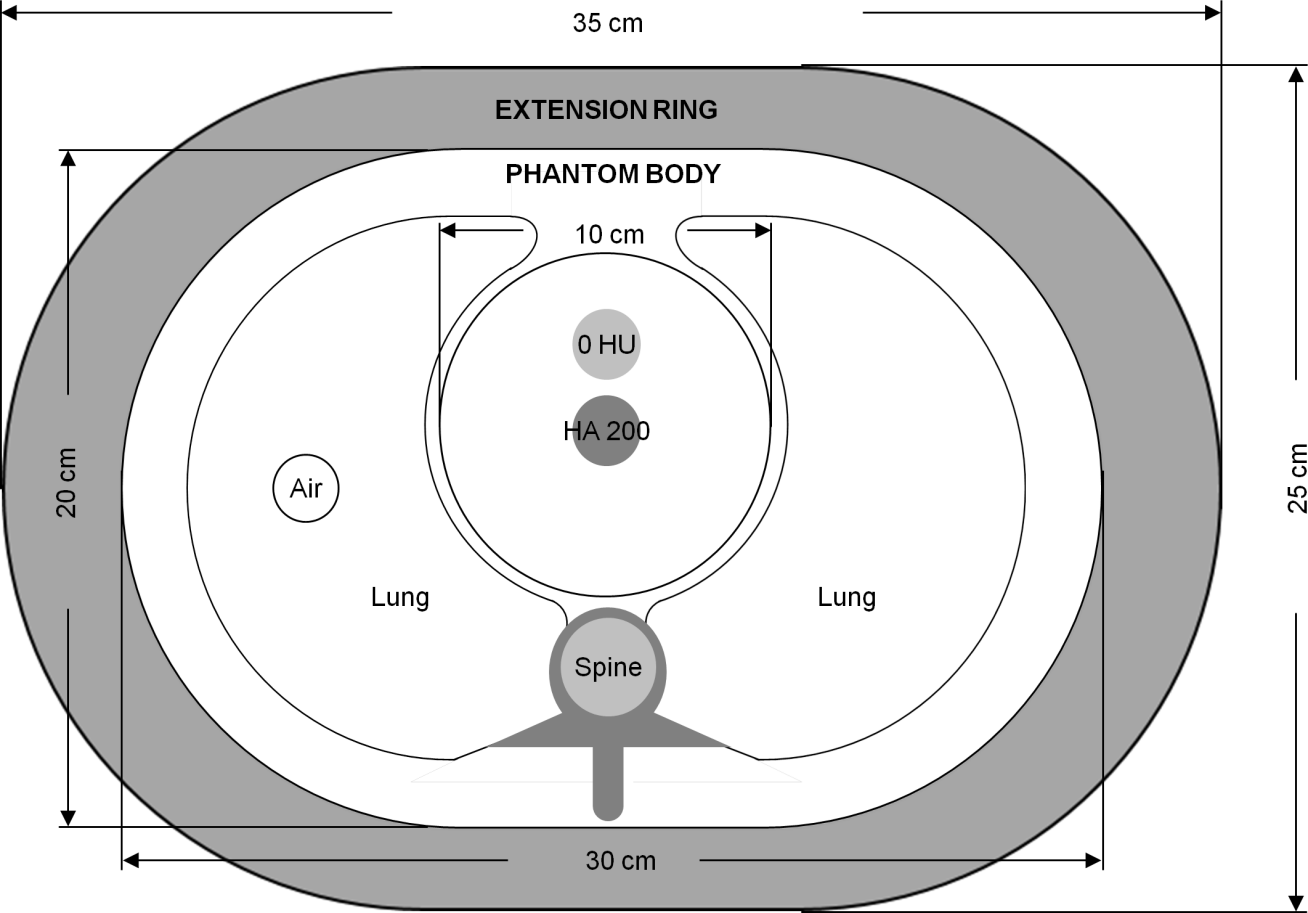
Illustration of the anthropomorphic chest phantom which consists of the phantom body and the extension ring.

An anthropomorphic chest phantom (QRM GmbH, Möhrendorf, Germany) which consist of phantom body and extension ring was used to simulate the setup for treatment planning of a lung cancer patient (Fig 6). The outer dimensions of the chest phantom are 35 cm × 25 cm in transverse plane and 15 cm in height. At the heart position of the phantom body is a calibration insert of 10 cm diameter which contains two homogeneous rods. One of them is made of water equivalent material, and the other one is made of calcium hydroxyapatite (CaHA) with density of 200 mg/cm^3^. The MDCT, CBCT, shading corrected CBCT and VMCT of the chest phantom were imported to the Eclipse planning station (Varian Medical Systems, Palo Alto, CA, USA) for treatment planning using 6 MV bilateral field arrangement.

## Results

According to the calibration step wedge experiment, the parameters a_i_, b_j_ c_i_, d_j_ in Eqs 1 and 2 were determined:

$$x_{A}=\frac{-0.135+4.814L-4.818H+0.0436-0.016LH+0.023H^{2}}{1-0.039L+0.046H}$$

$$x_{B}=\frac{0.922-14.786L+17.438H-1.639L^{2}+3.254LH-1.585H^{2}}{1+0.0252L-0.0172H}.$$

**Fig 7:**
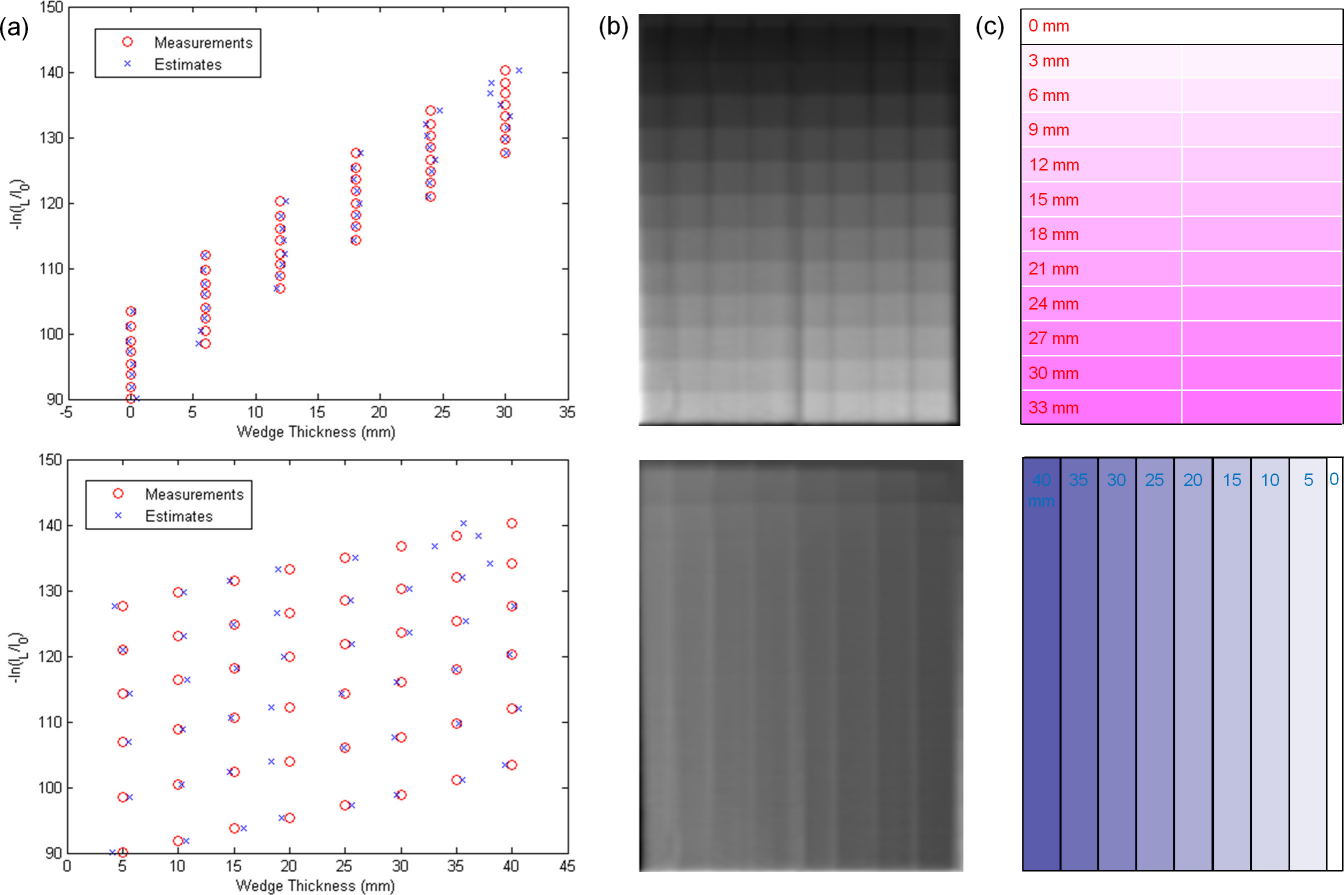
(a) Wedge thickness versus image intensity in low energy projection, (b) decomposed projections from basis material decomposition and (c) the corresponding illustrations for aluminum (top row) and acrylic (bottom row) step wedges.

**Fig 8:**
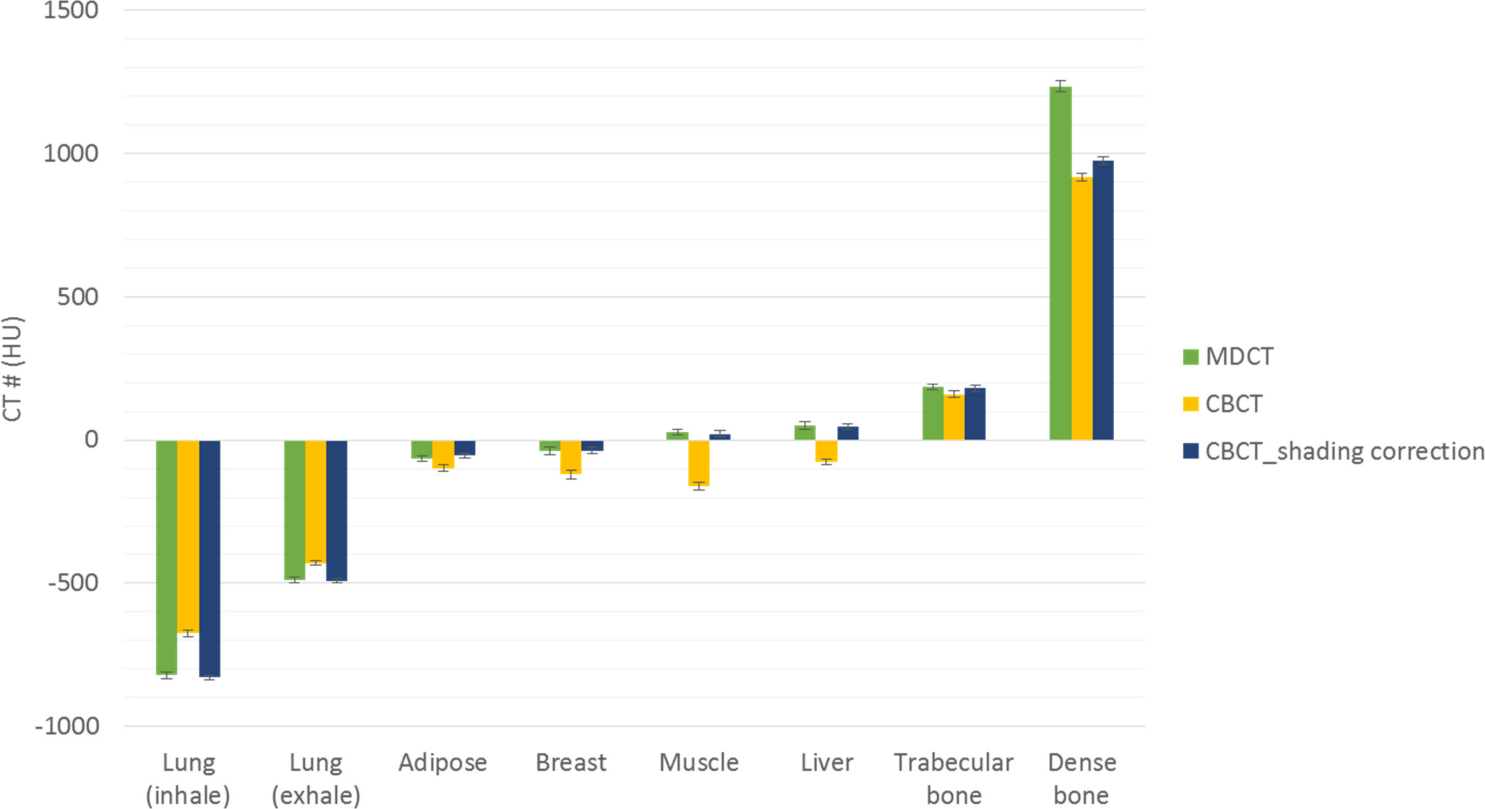
CT numbers of MDCT, original CBCT and shading corrected CBCT for the electron density phantom without titanium inserts.

**Fig 9:**
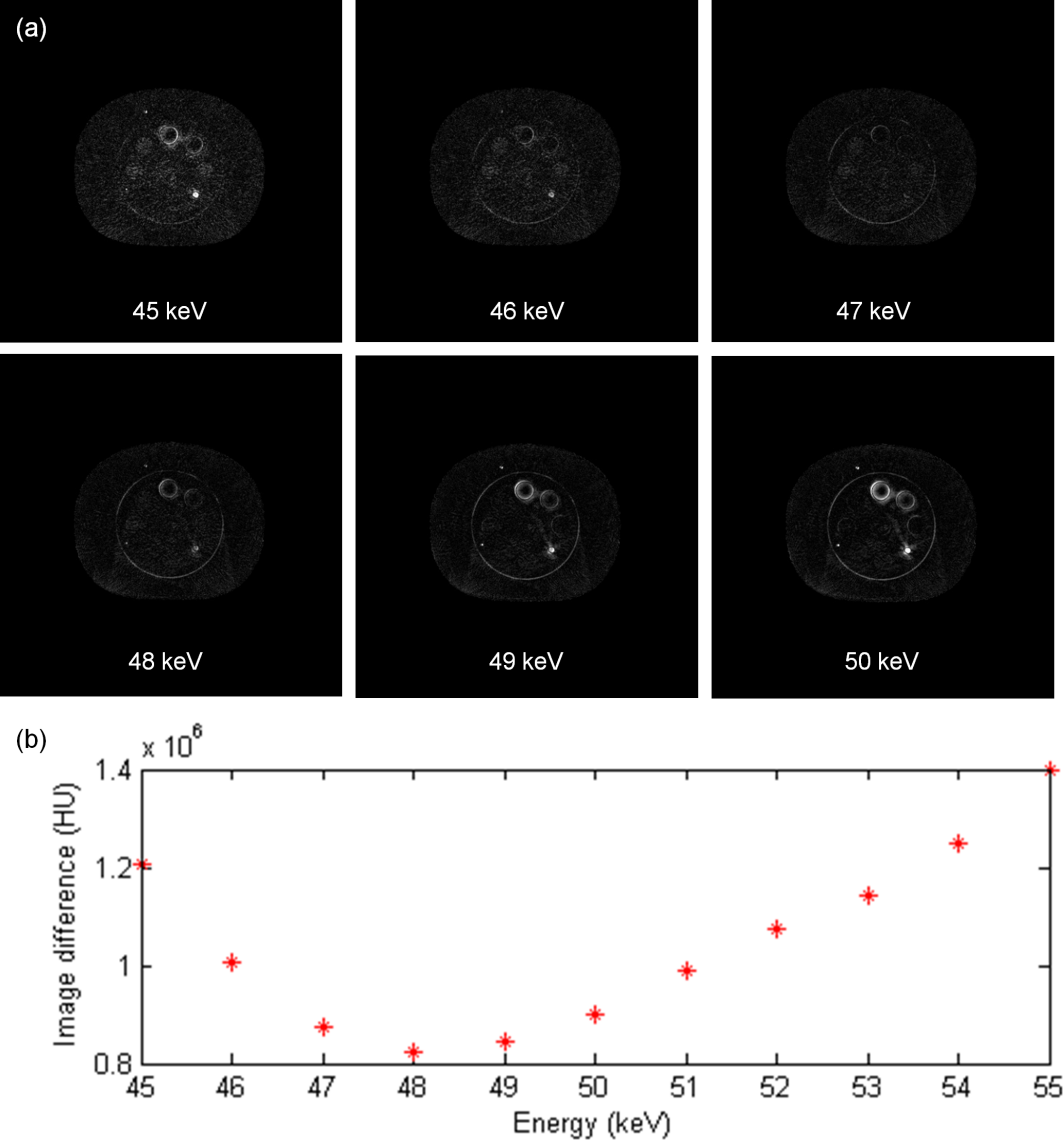
(a) Difference image between VMCT images synthesized using low-energy MDCT + standard MDCT and CBCT + MDCT at 45–50 keV for the electron density phantom without titanium inserts. (b) Total intensity of the difference image at different energies.

**Fig 10:**
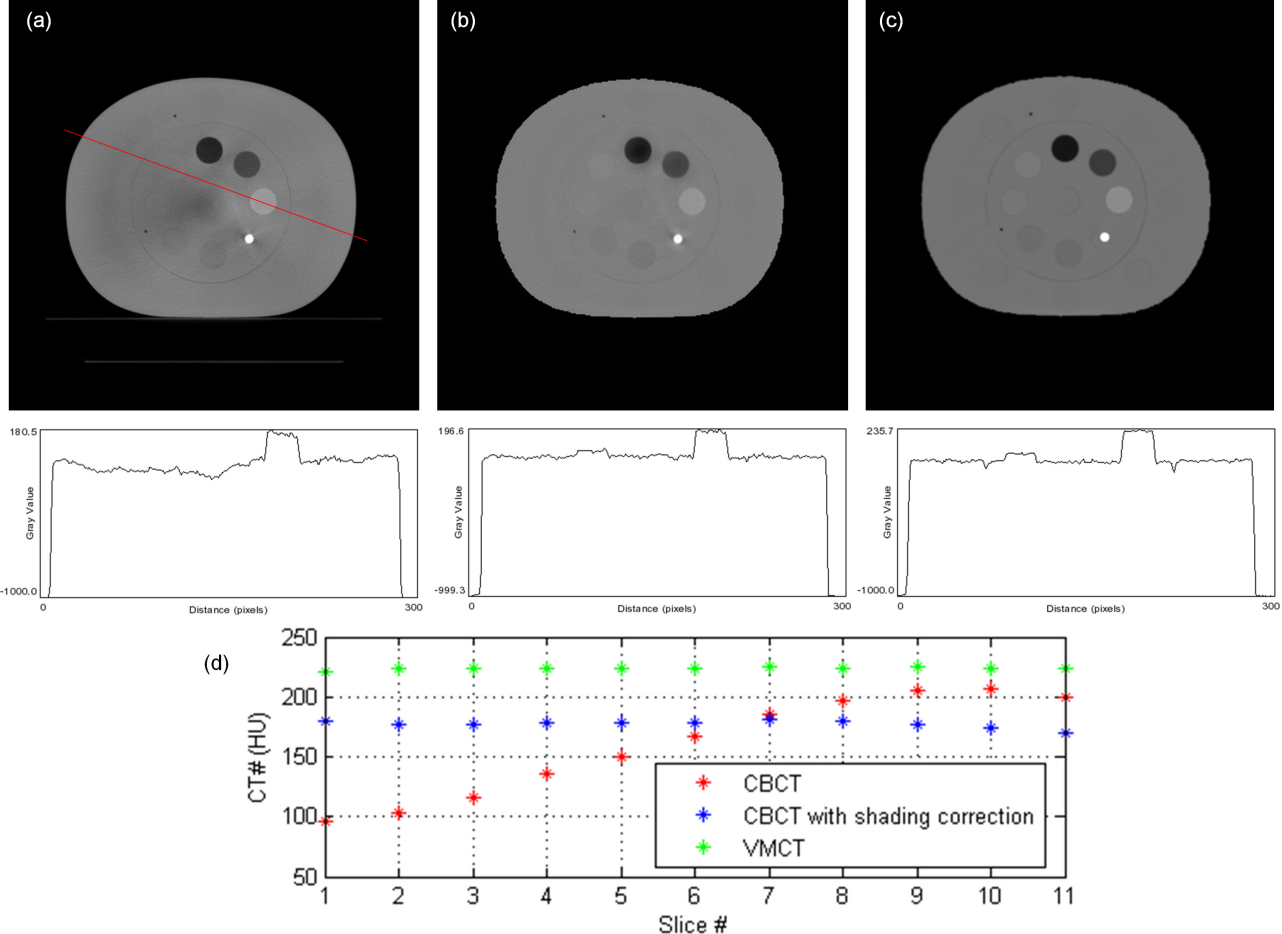
Axial image and intensity profile through the red line of (a) CBCT, (b) shading corrected CBCT and (c) VMCT at 48 keV synthesized using CBCT + MDCT for the electron density phantom without titanium inserts. (d) The mean CT number of the rod insert simulating trabecular bone in different axial slices.

**Fig 11:**
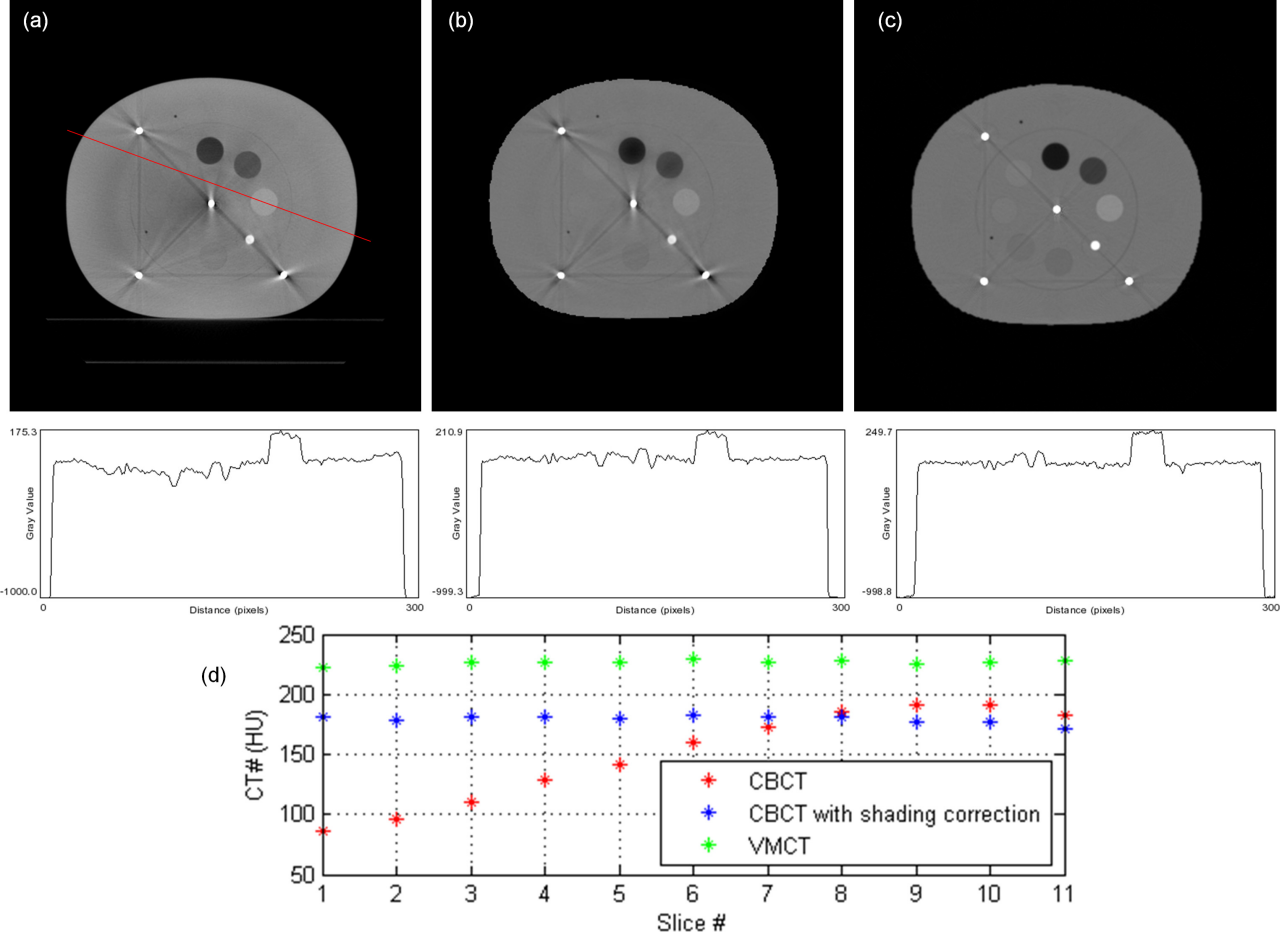
Axial image and intensity profile through the red line of (a) CBCT, (b) shading corrected CBCT and (c) VMCT at 48 keV synthesized using CBCT + MDCT for the electron density phantom with titanium inserts. (d) The mean CT number of the rod insert simulating trabecular bone in different axial slices.

**Fig 12:**
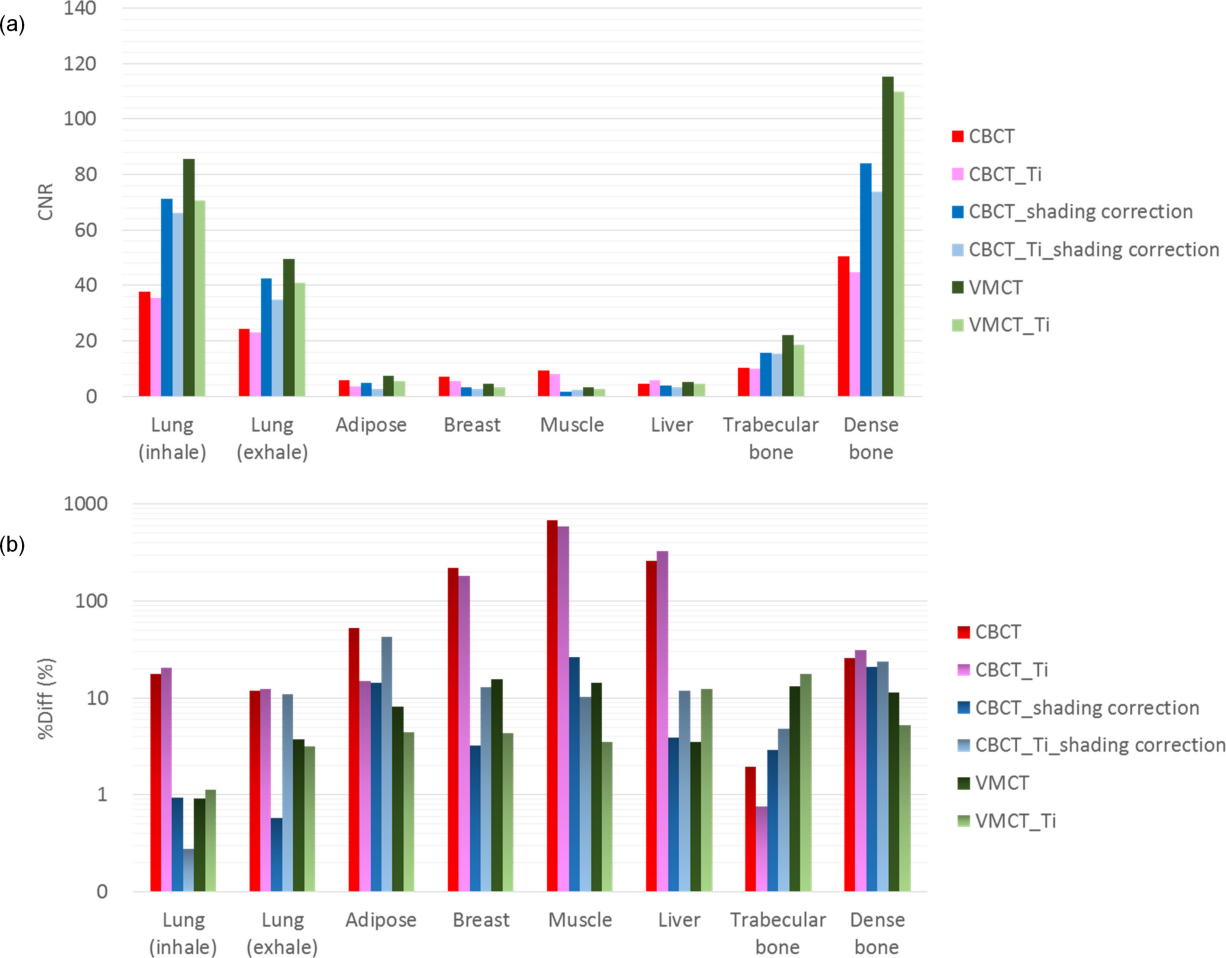
(a) CNR and (b) %Diff in CBCT, shading corrected CBCT and VMCT for the electron density phantom without and with titanium inserts.

**Fig 13:**
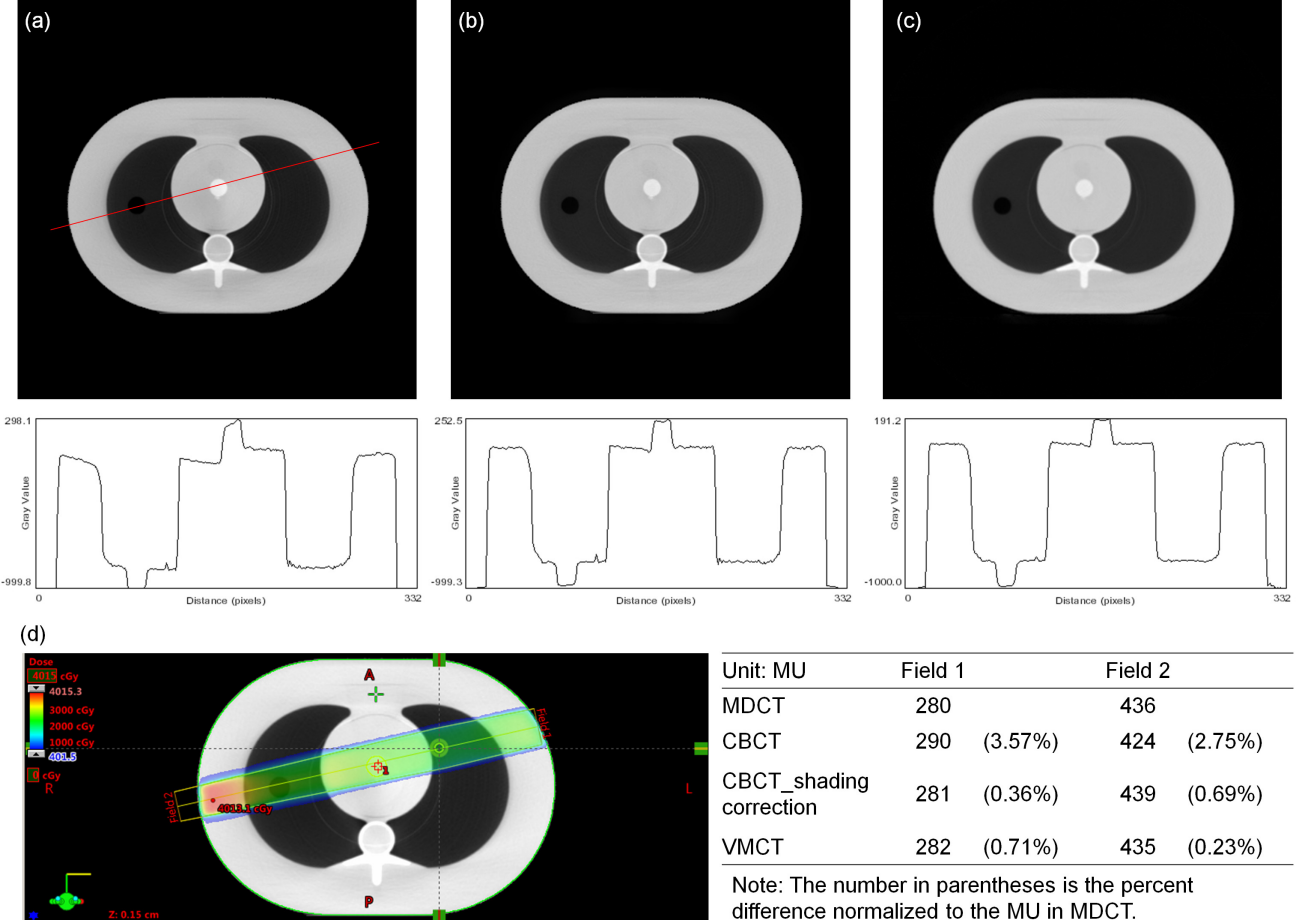
Axial image and intensity profile through the red line of (a) CBCT, (b) shading corrected CBCT and (c) VMCT at 48 keV synthesized using CBCT + MDCT for the chest phantom. (d) A phantom plan study (left) and the corresponding MU (right) for 6 MV photon beam irradiated bilaterally from the left side (field 1) and the right side (field 2).

Fig 7 demonstrates the comparison between the measured thickness and the estimated thickness obtained via the decomposition step for the calibration step wedge. Since the aluminum and acrylic step wedges were stacked in an orthogonal pattern, the photon beams passing through the aluminum step wedge with a specific step thickness may pass through the acrylic step wedge with thickness of 5, 10, 15, 20, 25, 30, 35 or 40 mm. Similarly, the photon beams passing through the acrylic step wedge with a specific step thickness may pass through the aluminum step wedge with thickness of 0, 6, 12, 18, 24 or 30 mm. The thickest wedge step results in the lowest photon intensity, i.e., the first row in Fig 7(A). The differences between measurements and estimates for the aluminum step wedge with thickness of 0, 6, 12, 18, 24, 30 mm are 0.24, 0.15, 0.23, 0.16, 0.29, 0.58 mm, respectively. The differences between measurements and estimates for the acrylic step wedge with thickness of 5, 10, 15, 20, 25, 30, 35, 40 mm are 0.80, 0.53, 0.24, 1.03, 0.64, 1.02, 0.91, 1.56 mm, respectively. Overall, the estimates show agreement with the measurements within 5%. The CT numbers of the rod inserts simulating different tissue materials in MDCT, original CBCT and shading corrected CBCT for the electron density phantom without titanium inserts are shown in Fig 8. It was found that the shading corrected CBCT agreed with the MDCT within 27%, while the original CBCT showed difference up to 680%. Fig 9 shows the absolute difference between VMCT images synthesized using low-energy MDCT + standard MDCT and those using CBCT + MDCT for the electron density phantom without titanium inserts. The smallest intensity of the difference images was found at 48 keV, i.e., the optimal energy of VMCT synthesized using CBCT + MDCT. Fig 10 shows the original CBCT, the shading corrected CBCT and the VMCT at 48 keV synthesized using CBCT + MDCT for the electron density phantom without titanium inserts and their intensity profiles in the transverse plane and in the axial direction. The corresponding results for the electron density phantom with titanium inserts are shown in Fig 11. Fig 12 summarizes the CNR and %Diff in CBCT, shading corrected CBCT and VMCT for the electron density phantom. The mean value of CNR for the electron density phantom without titanium inserts was 18.71, 28.40 and 36.65 in CBCT, shading-corrected CBCT and VMCT, respectively. The corresponding results for the electron density phantom with titanium inserts were 16.97, 25.15 and 31.96. The mean value of %Diff for the electron density phantom without titanium inserts was 158.32%, 9.16% and 6.49% in CBCT, shading-corrected CBCT and VMCT, respectively. The corresponding results for the electron density phantom with titanium inserts were 147.01%, 14.69% and 8.84%. With regards to the HU variation, the standard deviation within a uniform background of CBCT, shading-corrected CBCT and VMCT for the electron density phantom without titanium inserts was 74, 15 and 11 HU, respectively. The corresponding results for the electron density phantom with titanium inserts were 96, 31 and 19 HU. Fig 13 demonstrates the original CBCT, the shading corrected CBCT and the VMCT at 48 keV synthesized using CBCT + MDCT for the chest phantom and the monitor unit (MU) of their treatment plans. The MU difference between the treatment plan calculated using MDCT and those based on CBCT, shading corrected CBCT and VMCT was 6.32%, 1.05% and 0.94%, respectively.

## Discussion

Applications of CBCT to IGRT can be hampered by shading artifacts in the reconstructed images, especially when half-fan torso protocol is used to produce a field of view larger than 25 cm [9, 14]. The shading artifacts in CBCT images which leads to inaccuracies in the reconstructed CT numbers result from several nonidealities, including scattered radiation, beam hardening effects, detector lag, nonlinear detector gains, and the use of bow-tie filter. Among these factors, scatter is the dominant cause of shading artifacts. It has been reported by Marchant et al. that promising results can be obtained from CBCT corrected by using the MDCT-based shading correction [14]. According to our results, the shading correction improved the CNR from 17.84 to 26.78 and decreased %Diff from 152.76% to 11.93%. However, beam hardening artifacts caused by the rod insert simulating dense bone and metal inserts were observed in the shading corrected CBCT of the electron density phantom (Figs 10(B) and 11(B)). An x-ray beam hardens as the low energy components of the polychromatic spectrum suffer substantial attenuation when passing through the object. This may result in several artifacts such as cupping and streaking, which are quite common in CT images of patients who have permanent metallic implants such as dental fillings, hip or knee prostheses, cardiac pacemakers [18, 19]. Due to the high atomic number of dense materials, x-ray photons passing through these objects are highly attenuated and result bright and dark streaking artifacts in the reconstructed CT images. Occasionally, these artifacts are severe enough to degrade image quality and interfere with interpretation. Various beam hardening correction techniques have been proposed [15, 20, 21]. The dual-energy technique is a spectrum-based method which generates virtual monochromatic spectral images, so it should not suffer from beam hardening effects. Li et al. have proposed a dual-energy technique for CBCT used in IGRT [15]. Their method can effectively reduce beam hardening and metal artifacts, but it needs an additional CBCT scan besides the routine workflow. Since both MDCT and CBCT are performed in current clinical practice to help the clinician to make a better treatment plan, a workflow for synthesizing VMCT based on CBCT + MDCT was proposed and evaluated in this study.

Although the CT numbers in shading corrected CBCT show high agreement with MDCT results, differences still exist between VMCT images synthesized using CBCT + MDCT and those using low-energy MDCT + standard MDCT. For the electron density phantom without titanium inserts, the highest intensity in difference image was observed in the rod insert simulating dense bone, which could be due to the largest CT number difference between MDCT and shading corrected CBCT as compared to other tissue materials (Figs 8 and 9). In this study, the low-energy and high-energy projections used for synthesizing VMCT were obtained via forward projecting the reconstructed images. CT imaging is a way to visualize material linear attenuation coefficient, so the CT numbers in CBCT and MDCT with the same energy could be very close to each other if artifacts degrading image quality can be properly corrected. In manufacturer’s software, several data processing and correction steps have been applied during image reconstruction, such as geometry correction, beam hardening correction, scatter correction and so on [22, 23]. From our experience, the CT number of titanium insert which should be around 14291 HU at 52 keV and 10834 HU at 58 keV is fixed at 7000 HU in CBCT and is fixed at 2976 HU in either 90-kVp or 120-kVp MDCT. The on-board CBCT system utilizes 16-bit depth and a rescale intercept of -1000, so the CBCT images range from -1000 to 64535 in HU. On the other hand, the MDCT system utilizes 12-bit depth and a rescale intercept of -1024, so the MDCT images range from -1024 to 3071 in HU. HU data for dense materials may be truncated due to 12-bit depth saturating at high CT number. To combat this, scaling down of raw data and extending the CT to electron density curve to include metal data have been proposed [24]. However, it implies that the data processing and correction methods implemented in manufacturer’s software may change the data characteristics and thus affect the efficacy of VMCT synthesized using the proposed workflow, especially in dense materials. Although differences were observed between VMCT images synthesized using CBCT + MDCT and those using low-energy MDCT and standard MDCT, the shading artifacts and the streaking artifact observed in the original CBCT and the shading correct CBCT were reduced in the VMCT synthesized using CBCT + MDCT. Moreover, the image quality of VMCT at 48 keV was improved substantially in terms of CNR, %Diff and HU variation, demonstrating the efficacy of the proposed workflow for improving target detection and HU fidelity. The improved image quality in VMCT can contribute not only to daily patient setup but also adaptive dose delivery. Based on the treatment plan studies for the anthropomorphic chest phantom, the difference in MU between VMCT and MDCT is less than 1%, indicating that VMCT could be used for a possible plan adaptation in lung cancer cases.

This study demonstrates the efficacy of the proposed workflow on reducing the shading artifacts and the metal artifacts in torso CBCT scan. Compared with torso CBCT scan, the shading artifacts are scarcely observed in head CBCT scan. Contrary, the metal artifacts due to dental fillings are usually observed in head CBCT scan. The CBCT scanning protocols of head are different from those of torso in terms of scan mode (full scan vs. half scan), tube voltage (100 kVp vs. 125 kVp) and tube currents (3 options vs. a fixed mAs). The efficacy of the proposed workflow on reducing the metal artifacts in head CBCT scan was also investigated (data not shown). From our experience, substantial metal artifact reduction was observed even if low-dose CBCT protocol was used to synthesize VMCT for head scan (the CTDI_w_ of low-dose, standard and high-dose CBCT protocols of head are 1.5, 2.9 and 14.6 mSv, respectively). Several limitations to this study need to be acknowledged. First, the data acquisition, processing and reconstruction approaches can influence the study results. The protocol parameters used in this study are suggested by the manufacturers and are currently employed in many centers equipped with the same scanners. Additional studies assessing the proposed workflow for different MDCT or CBCT scanners will be needed and valuable. Second, all images were acquired with the electron density phantom and the anthropomorphic chest phantom. In this study, the MDCT images were resampled to the same voxel size as the CBCT and were then registered with CBCT images via affine transformation which used the gradient descent method for optimization. When the proposed workflow is translated to clinical use, it is expected that the accuracy of the registration step determines the performance of the proposed method. Challenges arise because patients have large organ deformation from the MDCT scanner to the treatment room. In clinical implementation, deformable registration should be applied to improve the geometry match between MDCT and CBCT. The efficacy of the proposed workflow on clinical patient data needs to be further investigated. Third, the computation efficiency of the proposed workflow in the current implementation is acceptable for off-line image-guided adaptive radiotherapy. For on-line or real time applications, efficient programming languages, parallel computation or hardware-based acceleration should be used to reduce the image processing time. Overall, this study has demonstrated the feasibility of using on-board CBCT and planning MDCT to realize VMCT for image quality improvement in on-board CBCT. Since MDCT and CBCT acquired in clinical IGRT workflow were used to synthesize VMCT, no additional CT scan is required. Hence, the proposed method provides effective image quality improvement without dose or scan time increases.

## Conclusion

This study demonstrated the feasibility of using on-board CBCT and planning MDCT to realize VMCT for image quality improvement in on-board CBCT. Based on our results, VMCT has the highest image quality in terms of CNR and HU fidelity, followed by the shading corrected CBCT and the original CBCT. Enhancement of image quality in on-board CBCT can contribute to daily patient setup and adaptive dose delivery, thus enabling higher confidence in patient treatment accuracy in radiation therapy. The research results present in this study should be able to provide a route to reach a high level of image quality for CBCT imaging used in radiation oncology.
